# Defenestration of Liver Sinusoidal Endothelial Cells: The Trigger of Liver Fibrosis

**DOI:** 10.3390/ph18060893

**Published:** 2025-06-14

**Authors:** Juntao Zhou, Jianqiao Wang, Lijuan Zhang, Chengliang Zhang, Cheng Tian

**Affiliations:** Department of Pharmacy, Tongji Hospital, Tongji Medical College, Huazhong University of Science and Technology, Wuhan 430030, China; zjt20250108@126.com (J.Z.); joanne1016213@163.com (J.W.); 14753065273@163.com (L.Z.)

**Keywords:** liver sinusoidal endothelial cells, fenestrae, liver fibrosis, oxidative stress, pathological angiogenesis, therapeutic strategies, capillarization

## Abstract

Liver fibrosis is a common pathological manifestation of various chronic liver diseases, distinguished by the excessive accumulation of the extracellular matrix. If unresolved, liver fibrosis can progress to cirrhosis or hepatocellular carcinoma. Fenestrae are important structures of liver sinusoidal endothelial cells (LSECs) regulating hepatic substance exchange, immune response and hemodynamics. The loss of this structure is usually accompanied by dysfunction of LSECs, which disrupts normal liver physiology by impairing hepatic substance exchange, compromising liver microcirculation, and activating hepatic stellate cells (HSCs). This cascade of events ultimately contributes to the onset and development of liver fibrosis. Oxidative stress, impairment of the NO signaling pathway, actin–myosin complex remodeling and pathological angiogenesis are considered to be the main mechanisms underlying LSEC defenestration. Recently, research on the treatment of LSEC defenestration has made notable progress, and findings suggest a potential value in the application of anti-fibrotic therapies. This article expounds the important correlation between defenestration of LSECs and liver fibrosis, while also reviews therapeutic agents and approaches targeting this pathological process.

## 1. Introduction

Liver fibrosis, a common pathological manifestation of numerous chronic liver diseases, is distinguished by the excessive accumulation of the extracellular matrix (ECM) [1,2]. The main strategies of anti-fibrotic therapy include protecting hepatocytes, inhibiting the activation of hepatic stellate cells (HSCs) and regulating immune response. However, no specific drug is able to effectively reverse liver fibrosis, leaving liver transplantation as the sole curative therapy for advanced cirrhosis [2,3].

Liver sinusoidal endothelial cells (LSECs), located in the liver sinusoids, are the first cells that contact with the liver’s blood flow and are among the initial cells affected by external etiological factors during the progression of liver fibrosis [4,5]. LSECs not only play a key role in substance exchange but also participate in regulating the immune surveillance and substances clearance of the liver. These cells can recognize and phagocytose the bioactive substances produced by bacteria, viruses and other entities in the bloodstream, in order to help maintain the immune homeostasis of the liver [6]. Moreover, LSECs are essential for regulating liver angiogenesis and preserving the quiescence of HSCs [7].

Unlike other endothelial cells, LSECs lack the basement membrane and septa, featuring unique pore structures called fenestrae in their membranes. These fenestrae are arranged to form a “sieve plate” structure, facilitating efficient material exchange between hepatocytes and the blood [8]. And they are indispensable for LSECs in performing normal functions. LSEC capillarization, also known as LSEC defenestration, will lead to LSEC dysfunction, thereby inducing or accelerating the progression of liver fibrosis [9,10]. This review aims to explore the role of LSEC’s fenestrae in liver fibrosis, the mechanisms of LSEC defenestration, and related therapeutic drugs.

## 2. Physiological Functions of Fenestrae of LSECs

Fenestrae are small pores found on the membranes of LSECs, typically measuring 50–200 nm in diameter and lacking a basement membrane. This unique structure facilitates the transfer of substances from the bloodstream into the space of Disse, playing an important role in regulating the liver microenvironment [9,11].

### 2.1. Substance Exchange

The presence of fenestrae enables LSECs to effectively carry out filtration and the substance exchange process, forming a functional barrier with high permeability between the Disse cavity and blood [12]. The permeability of this barrier is affected by the porosity of fenestrae, which selectively regulates the transport of substances based on their size and properties. This barrier also allows bidirectional passive transport of substances including water, oxygen, micronutrients, macronutrients, lipoproteins and cholesterol, while preventing the passage of larger molecules and cellular components. In addition, the efficient transport of certain drugs, such as statins and insulin, are also modulated by this specific barrier [9,11].

### 2.2. Immune Regulation

Through promoting the recruitment of immune cells from the circulation to liver parenchymal cells, the fenestrae of LSECs enable immune cells to cross the vascular barrier and enter the liver tissue more efficiently [13]. Enlarged fenestrae and increased vascular permeability lead to the abnormal migration and activation of immune cells, thereby aggravating liver inflammation and promoting micro-thrombosis [14]. Additionally, the fenestrae also mediate hepatic immune tolerance. Liver-resident lymphocytes and circulating native CD8^+^ T cells can pass through the fenestrae via cytoplasmic extension, enabling direct contact with hepatocytes. This unique interaction is of great significance in mediating liver immune tolerance [15]. Furthermore, the fenestrae help LSECs to endocytose rapidly and efficiently, allowing for the effective monitoring and clearance of bioactive substances, particularly immune-related molecules, such as immune complexes and bacterial endotoxins, from the bloodstream, thereby supporting immune homeostasis [7].

### 2.3. Liver Hemodynamics Regulation

The fenestrae are crucial for maintaining efficient substance exchange and oxygen delivery within the liver. Under physiological conditions, there is no significant osmotic and hydrostatic pressure gradient across the normal liver sinusoids, which allows small molecules and gases to diffuse freely through the fenestrae [8]. Notably, the presence of fenestrae in LSECs enables portal venous pressure to remain stable and relatively low, even during significant fluctuations in blood flow, such as after a meal. Variations in the size and number of fenestrae can also significantly alter hepatic hemodynamics, thereby modulating portal venous pressure and influencing the progression of liver disease [13,16].

## 3. LSEC Defenestration

Liver sinusoidal endothelial cells are the primary cells that interact with liver blood flow and toxic stimuli in the blood. During the progression of liver fibrosis, LSECs exhibit a marked increase in the expression of CD31, CD34, and molecules such as von Willebrand factor (VWF). Concurrently, these cells lose their characteristic fenestrae, develop basement membranes, and ultimately transform into a continuous endothelium. This pathological process is called liver sinusoidal endothelial cell capillarization or defenestration, which significantly contributes to liver fibrosis [7,17]. Notably, early removal of the underlying etiology has been demonstrated to reverse this pathological change [18].

### 3.1. Influencing Factors for LSEC Defenestration

The morphology of fenestrae, including diameter, porosity, and quantity, is influenced by pathophysiological conditions, such as blood flow shear stress and external stimuli factors, like diet, drugs and toxins.

Blood flow shear stress: Shear stress is instrumental in regulating the morphology of fenestrae, with the transcription factor Krüppel-like factor 2 (KLF2) serving as a critical regulator of phenotype of LSECs. Notably, changes in blood flow shear stress affect the expression level of KLF2 in LSECs. Such changes cause morphological alterations in the fenestrae of LSECs, thereby compromise their anti-inflammatory and anti-fibrotic abilities. These dysfunctions ultimately accelerate the progression of liver disease [8,19].

Diet: A high-fat diet significantly elevates the level of oxidatively modified low-density lipoprotein (ox-LDL), a product of lipid oxidation that directly damages LSECs and induces fenestrae loss [20]. In addition, high protein or high carbohydrate intake is linked to a decrease in both the diameter and density of fenestrae in LSECs [21].

Drugs and poisons: A variety of drugs and poisons can induce LSEC defenestration by promoting the overproduction of reactive oxygen species (ROS). Acetaminophen and low-dose arsenic initially damage LSECs via inducing oxidative stress, and further lead to LSEC defenestration and liver sinusoidal microcirculation disorder. Additionally, excessive alcohol exposure has been found to reduce the number of fenestrae while increasing their diameter, resulting in the reduction of overall porosity. However, the specific mechanism underlying this phenomenon has not been fully elucidated [22,23].

**Figure 1:**
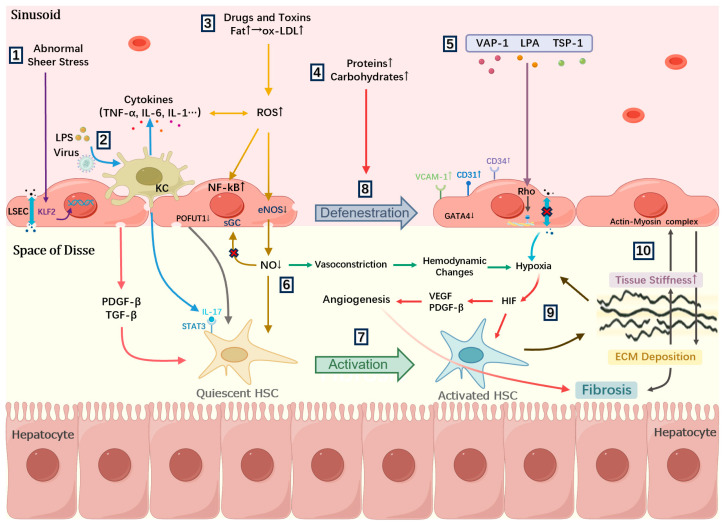
The mechanisms of LSEC defenestration and the role of defenestrated LSEC in liver fibrosis. 1–6: LSEC defenestration could be induced by various factors. (1) Abnormal shear stress can affect the morphology of fenestrae by affecting the expression level of KLF2 in LSECs. (2) LPS and virus mediate inflammatory response by activating KCs, which subsequently secrete pro-inflammatory cytokines, such as TNF-α, IL-6 and IL-1. This activation upregulates the NF-κB signaling pathway, leading to the loss of LSEC fenestrae. (3) Excessive ROS generation caused by toxins, drugs, ox-LDL and inflammatory response can directly damage LSECs, activate the NF-κB signaling pathway, and influence the synthesis and utilization of NO, ultimately leading to LSEC defenestration. (4) High protein or high carbohydrate intake is associated with reduction in both the diameter and number of fenestrae in LSECs. (5) Bioactive Substances like TSP-1, VAP-1 and LPA can activate the Rho signaling pathway, which enhances the contractile capacity of the actin–myosin complex, ultimately leading to the contraction of fenestrae. (6) The suppressed eNOS activity results in significant dysfunction of NO, which subsequently disrupts the sGC signaling pathway. 7–9: Defenestrated LSECs mediate liver fibrosis through multiple pathways. (7) The activation of HSCs is regulated by various bio-mediums, including TGF-β, PDGF, IL-17, fibrinogen and NO. During the process of LSEC phenotypic transition, elevated levels of TGF-β and PDGF-β, along with impaired NO signaling, contribute to HSC activation. Additionally, IL-17 secreted by activated KCs and fibrinogen upregulation resulting from POFUT1 loss can also promote HSC activation and type I collagen production. (8) Defenestration is associated with upregulation of VCAM-1, CD31 and CD34, loss of GATA4 signaling, reduced fenestrations, basement membrane synthesis and substance exchange dysfunction, leading to glycol-metabolic and lipo-metabolic disorders. (9) Hypoxia, driven by abnormal oxygen distribution and hemodynamic disturbances, promotes angiogenesis and activates HSCs via the HIF-1α signaling pathways. ECM deposition also aggravates hypoxia severity, establishing a vicious cycle that exacerbates pathological progression. (10) Increased tissue stiffness triggers actin cytoskeletal remodeling, leading to the loss of LSEC fenestrae and subsequently promoting ECM deposition.

Other factors: Dysbiosis of intestinal microbiota or impairment of the intestinal barrier can facilitate the translocation of lipopolysaccharides (LPS) into the bloodstream. Elevated LPS level initiates inflammatory response by activating Kupffer cells (KCs), which subsequently causes the loss of fenestrae in LSECs (Figure 1) [5,24]. Furthermore, infections with the hepatitis C virus (HCV) or Schistosoma japonicum also promote excessive production of ROS through inflammatory mechanisms, resulting in morphological alterations of fenestrae [25].

### 3.2. Impact of LSEC Defenestration on Liver Physiology

#### 3.2.1. Substance Exchange Disorder and Metabolic Dysfunction

The fenestrae of LSECs function as essential channels for the bidirectional exchange of substances between the blood and hepatocytes in the hepatic microcirculation. During the process of LSEC defenestration, however, there is a reduction in both the diameter and number of fenestrae, along with the gradual formation of a continuous basement membrane. These pathological alterations significantly hinder the substance exchange between the blood and hepatocytes, leading to glucose metabolism and lipid metabolism disorders [26,27]. Moreover, LSEC defenestration also severely disrupts the delivery of oxygen and creates a hypoxic environment in the liver [28]. In addition, LSEC defenestration significantly diminishes the capacity of the liver to metabolize drugs. Reducing the transport efficiency of drugs into hepatocytes may also result in an elevated concentration of certain drugs in the bloodstream, thereby increasing their toxicity risk. As for therapeutic agents targeting hepatocytes, their efficacy will be compromised due to reduced transport efficiency [29].

#### 3.2.2. Hepatic Hemodynamic Injury

LSEC defenestration not only reduces the permeability of hepatic sinusoids but also impedes the normal blood flow within the liver, leading to increased portal vein pressure. Furthermore, LSECs play a critical role in regulating vascular tone by secreting nitric oxide (NO). However, during the process of LSEC defenestration, the ability of LSECs to secrete NO is suppressed, resulting in sinusoidal constriction. This narrowing of the sinusoidal lumen increases intrahepatic blood flow resistance, thereby aggravating the severity of hepatic microcirculatory disorder [10,30]. These pathological alterations constitute an important foundation for hepatic hemodynamic injury.

#### 3.2.3. Impairment of Liver Regeneration Function

LSEC defenestration also impairs the liver’s regenerative capacity. In this process, the secretion levels of signaling molecules, mainly produced by LSECs and closely associated with hepatocyte proliferation, such as hepatocyte growth factor (HGF) and Wnt family member 2α (Wnt2α), are significantly decreased, which markedly compromise the regeneration ability of hepatocytes. This disruption further weakens the self-healing and adaptive ability of the liver, making it difficult to effectively cope with various damaging factors [31].

### 3.3. Main Mechanisms of LSEC Defenestration

The specific mechanisms underlying LSEC defenestration have not been fully elucidated. However, current studies suggest that oxidative stress injury, impairment of the NO signaling pathway, actin–myosin complex remodeling, and pathological angiogenesis are the primary contributors to this process. These mechanisms interact with one another, ultimately leading to the loss of fenestrae in LSECs.

#### 3.3.1. Oxidative Stress Injury

Oxidative stress is widely regarded as a crucial mechanism underlying the structural alterations of LSEC fenestrae [32]. Due to their relatively low antioxidant enzyme activity and limited antioxidant defenses, LSECs are particularly sensitive to oxidative stress [33]. Various injurious stimuli can induce excessive generation of ROS, which in turn activates the NF-κB signaling pathway and upregulates the expression of pro-inflammatory factors, ultimately disrupting the normal structure of fenestrae [34]. Studies have indicated that acetaminophen induces oxidative stress damage by increasing superoxide anions, leading to disruptions in the fenestration structure of LSECs [22]. In addition, ox-LDL upregulates lectin-like ox-LDL receptor 1 (LOX-1) expression in LSECs, activates the ROS/NF-κB signaling pathway, and induces LSEC defenestration [20]. It is also reported that ROS-induced LSEC defenestration is related to the oxidation and destabilization of spectrin, which connects the cell membrane to the fenestra-associated cytoskeleton [32]. In addition, oxidative stress also impacts the autophagic function of LSECs. Luo et al. found that oxidative stress induced by aldosterone activates the AMPK signaling pathway through caveolin-1 related selective autophagy, while inhibiting NO-dependent signaling pathways, ultimately resulting in the defenestration of LSECs [35]. Consequently, inhibiting the autophagy of LSECs may become a potential therapeutic strategy to prevent LSEC capillarization and liver fibrosis.

#### 3.3.2. Impairment of the NO Signaling Pathway

NO maintains the normal morphology and function of LSECs by regulating vascular tone, blood flow, and intracellular signal transduction [36]. However, under various pathogenic stimuli, the synthesis and utilization of NO are impaired, ultimately leading to LSEC defenestration. For instance, ROS react with NO to generate reactive nitrogen species (RNS), thereby decreasing the bioavailability of NO. Simultaneously, ROS impair the function of endothelial nitric oxide synthase (eNOS) and interfere with the soluble guanylate cyclase (sGC)/cGMP/PKG signaling pathway, ultimately leading to the loss of fenestrae in LSECs [8,37]. Furthermore, portal hypertension and structural remodeling of the hepatic sinusoids alter the shear stress experienced by LSECs, thereby disrupting KLF2 expression and eNOS-mediated NO production. The ensuing NO deficiency consequently leads to LSEC defenestration [8]. Additionally, activation of the Notch signaling pathway in transgenic mice downregulates both eNOS expression and its phosphorylation levels, while knockout of the specific proteins bone morphogenetic protein 9 (BMP9) and protein O-fucosyl-transferase 1 (POFUT1) exerts the same effect, ultimately inducing LSEC defenestration [31,38,39].

#### 3.3.3. Actin–Myosin Complex Remodeling

As a cytoskeletal component of LSECs, the actin–myosin complex also plays an important role in the alterations of fenestrae. The Rho GTPases regulate this complex by influencing the polymerization state of myosin heavy chains and F-actin, thereby directly controlling the contraction and relaxation of fenestrae [40]. Relevant studies have shown that thrombospondin-1(TSP-1), vascular adhesion protein-1 (VAP-1), and lysophosphatidic acid (LPA) can activate the Rho signaling pathway and promote the phosphorylation of the myosin light chain (MLC) via Rho-Associated Kinase (ROCK). This process not only enhances the contractile capacity of the actin–myosin complex, but also increases F-actin stress fibers, ultimately leading to the contraction of fenestrae [40,41,42]. Additionally, increased mechanical stress can induce actin–myosin complex remodeling by activating the p38/MK2 pathway, eventually resulting in the loss of fenestrae of LSECs [28]. It is worth noting that the upregulated expression of Midkine (MK) can directly contribute to the remodeling of actin and cytoskeleton by activating the MK/Integrin α6/Src signaling pathway during the development of chronic liver disease [43].

#### 3.3.4. Pathological Angiogenesis

Pathological angiogenesis also drives LSEC defenestration. The development of this process is closely related to various pathophysiological conditions, including hemodynamic injury, inflammatory response, hypoxia and ECM deposition. These factors promote abnormal proliferation of blood vessels through the upregulation of vascular endothelial growth factor (VEGF). For instance, both the altered shear stress caused by hemodynamic injury and inflammatory response can converge to upregulate the VEGF signaling pathway [44,45]. Under hypoxic conditions, the increased expression of hypoxia inducible factor-1α (HIF-1α) also promotes the secretion of VEGF and amplifies this pathway, ultimately driving pathological angiogenesis and leading to LSEC defenestration [46]. Furthermore, platelet-derived growth factor (PDGF), another hypoxia-induced mediator, can synergize with VEGF to promote angiogenesis and induce defenestration of LSECs, which is closely associated with pathological vascular remodeling and liver fibrosis [47]. Notably, in a metabolic-dysfunction-associated steatohepatitis (MASH)-induced mouse model of liver fibrosis, the activation of the NF-κB signaling pathway and elevated angiopoietin-2 expression are found to precipitate pathological angiogenesis and LSEC defenestration [48].

## 4. Relationships Between LSEC Defenestration and Liver Fibrosis

### 4.1. LSEC Defenestration as a Critical Event in Liver Fibrosis

Several studies suggest that LSEC defenestration is a crucial event in early liver fibrosis, and restoring the phenotype of LSECs can effectively prevent or even reverse the development of liver fibrosis. The importance of LSEC defenestration in promoting fibrogenesis has been confirmed in both chemically induced and metabolic models of liver fibrosis. In mouse models of liver fibrosis induced by dimethyl-nitrosamine (DMN), carbon tetrachloride (CCl_4_), and thioacetamide (TAA), LSEC defenestration has been observed during the early stages of liver fibrosis [7,49,50]. Similarly, mice fed with methionine-choline-deficient (MCD) diet have also shown significant structural changes in fenestrae, characterized by the compression of sinusoidal pores, disorganized pore structures, the formation of multiple interconnected blood vessels and the occurrence of blind-ended dilations. Notably, these morphological alterations precede the onset of liver fibrosis [34]. Furthermore, the loss of GATA-binding protein 4 (GATA4), an essential transcription factor in LSEC development, also directly leads to the loss of fenestrae and liver fibrosis [51]. Additionally, clinical research has also demonstrated the presence of LSEC defenestration in human liver fibrosis and its significant role in driving the progression of liver fibrosis. In patients with primary biliary cirrhosis (PBC), LSECs undergo capillarization, along with the formation of basement membrane. This transformation is deemed to facilitate the progression of liver fibrosis [52].

### 4.2. Mechanism of LSEC Defenestration Promoting Liver Fibrosis

In the progression of liver fibrosis, activated HSCs represent the predominant source of ECM. Under physiological conditions, LSECs are essential for preserving the quiescent state of HSCs [53,54,55]. Nevertheless, during the onset and development of chronic liver disease, various injurious factors trigger the defenestration of LSECs, which subsequently results in their loss of the ability to suppress the activation of HSCs [56]. We have created a mechanism diagram to better illustrate the related content (Figure 1).

#### 4.2.1. Engendering a Hypoxic Environment in the Liver

The changes in LSEC phenotype result in the narrowing of the hepatic sinusoidal lumen and a subsequent decreased blood flow, significantly impacting hepatic hemodynamics [30]. These structural and functional alterations also disrupt normal oxygen distribution, shaping a hypoxic microenvironment in the liver and triggering metabolic dysfunction. Moreover, hypoxic activity stimulates angiogenesis and promotes HSCs activation through HIF-1α signaling pathway, contributing to excessive deposition of ECM. This abnormal matrix deposition further exacerbates the degree of LSEC defenestration, intensifying the hypoxic microenvironment and establishing a vicious cycle that accelerates the progression of liver fibrosis [47].

#### 4.2.2. Dysfunction of NO Synthesis

The phenotypic transformation of LSECs significantly impairs their ability to synthesize nitric oxide (NO). The deficiency of NO also diminishes the inhibitory role of LSECs in HSC activation. In turn, activated HSCs accelerate the development of liver fibrosis by excessive production of ECM [17].

#### 4.2.3. Overproduction of Profibrotic Factors and Pro-Inflammatory Factors

LSEC defenestration can markedly upregulate the expression levels of pro-fibrotic factors, including transforming growth factor-β (TGF-β) and platelet-derived growth factor (PDGF). Additionally, during the process of LSEC defenestration, the abnormally secreted pro-inflammatory factors, such as tumor necrosis factor-α (TNF-α), interleukin (IL)-1, IL-6, IL-17 and chemokine ligand 2 (CCL2), mediate liver inflammation by activating Kupffer cells (KCs) [57,58]. These pathological alterations can mediate HSC activation and further deteriorate the liver microenvironment by inducing the formation of basement membrane, ultimately driving the progression of liver fibrosis [59].

## 5. Research Progress on Restoration of Fenestrae of LSECs in the Treatment of Liver Fibrosis

LSEC defenestration is a common pathological phenomenon in the process of chronic liver disease and liver fibrosis. Recent studies have shown that restoring the fenestrae of LSECs can effectively maintain the resting state of HSCs and reverse liver fibrosis [7]. The related therapeutic drugs, along with their mechanisms for treating LSEC defenestration and liver fibrosis, are listed in Table 1, Table 2, Table 3 and Table 4.

### 5.1. Drugs to Alleviate Oxidative Stress Damage

Oxidative stress is primarily driven by excessive ROS generated by mitochondria, with NADPH oxidase (NOX) also serving as a significant source of ROS [23]. Studies suggest that reducing or eliminating ROS is a critical strategy to alleviate oxidative stress-induced damage to LSECs, which in turn helps treat liver fibrosis. Additionally, suppressing the inflammatory response caused by oxidative stress can also restore the fenestrae of LSECs (Table 1).

Resveratrol is a polyphenolic compound, characterized by its 3,5,4′-trihydroxy-trans-stilbene structure, which contributes to its ability to interact with various biological systems [60]. It has significant anti-inflammatory and anti-oxidative effects. By inhibiting NOX2 activity, resveratrol can effectively alleviate the damage of LSEC caused by oxidative stress, thus preventing the degeneration of LSEC fenestrae and further inhibiting the development of liver fibrosis [61]. Despite its potent biological activities, resveratrol’s short half-life and low bioavailability pose challenges. Fortunately, strategies such as nano-formulations are being explored to solve these problems [62].

N-acetylcysteine (NAC) is a commonly used antioxidant. The direct scavenging efficiency of NAC against primary reactive oxygen species (such as hydrogen peroxide, superoxide anions, etc.) is relatively low because its thiol group (–SH) has a comparatively low dissociation degree at physiological pH. However, NAC can exert indirect anti-oxidative effects through multiple pathways, such as by promoting the production of acyl thiols (R-S-SH) in cells and replenishing glutathione (GSH) [63]. In addition, NAC has anti-inflammatory effects, showing protective effects against CCl_4_-induced liver fibrosis [64]. NAC can also alleviate oxidative stress damage, ultimately preserving the structural integrity of LSEC fenestrae and mitigating liver fibrosis [22,65,66].

Curcumin, a natural polyphenol isolated from turmeric, has been documented to possess anti-oxidative and anti-cancer activities. Curcumin alleviates oxidative stress-mediated inflammation by inhibiting the NF-κB pathway, thereby promoting the reestablishment of the fenestrae structure of LSECs and mitigating liver fibrosis [67]. However, Curcumin’s poor aqueous solubility limits its bioavailability when administered orally. To address this, curcumin nanoparticles have been developed, which improve its physicochemical properties by reducing particle size and forming an amorphous state with hydrogen bonding [68]. In addition, structural modifications, such as the incorporation of heterocyclic groups in curcumin, can also improve its solubility and bioavailability [69].

### 5.2. eNOS and sGC Receptor Agonist

NO exerts a crucial role in maintaining the phenotype of LSECs by activating the sGC/cGMP/PKG signaling pathway. Impairment of the NO-dependent pathway can lead to LSEC dedifferentiation and liver fibrosis, while its activation can reverse these effects and support liver health [56] (Table 2).

Tofogliflozin is a sodium–glucose cotransporter 2 (SGLT2) inhibitor and is primarily used for the treatment of diabetes. Due to the fact that LSECs express high levels of SGLT2, tofogliflozin exhibits a degree of targeting. Studies have shown that tofogliflozin significantly attenuates liver fibrosis by increasing the activity of LSEC anti-oxidative enzymes, upregulating eNOS expression and its phosphorylation levels, and further restoring the LSEC phenotype in reducing collagen deposition [70].

Sulodexide is a heparinoid compound that combines the properties of its components, heparin and dermatan sulfate. With a molecular weight of less than 8000, it has high bioavailability [71]. It is also reported that sulodexide has anticoagulant, fibrinolytic, and anti-inflammatory effects. Furthermore, it alleviates oxidative stress damage by inhibiting the expression of NOX4 and NOX5 [72]. A recent study indicates that it plays a significant role in treating liver fibrosis by upregulation of eNOS expression to restore LSEC fenestrae, ultimately alleviating the progression of fibrosis [73].

Atorvastatin is a lipid-lowering medication, and recent research indicates that it possesses novel properties, such as upregulating eNOS, as well as anti-oxidative and anti-fibrotic effects. Furthermore, animal experiments have shown that atorvastatin can reduce NOX-1 expression in mice, suggesting potential benefits in combating hepatic fibrosis [74]. In addition, Atorvastatin increases phosphorylation of eNOS in liver tissues and LSECs, ultimately reversing liver fibrosis. However, atorvastatin has poor aqueous solubility due to its structure, consisting of three phenyl rings, which limits its bioavailability [75]. Fortunately, encapsulating atorvastatin with neutrophil liposomes can resolve this issue and enhance targeting of inflamed areas in the liver [76].

Metformin is a widely used oral medication primarily for managing type 2 diabetes and weight management. A study suggests that metformin can treat hepatic fibrosis by inducing apoptosis of HSCs [77]. In addition, it can upregulate the expression of eNOS in LSECs, thereby increasing NO levels in vivo. Such enhancement helps preserve the normal phenotype and physiological functions of LSECs, improves hepatic circulation, and further alleviates liver fibrosis [78].

Additionally, sGC receptor agonists, such as riociguat, a sGC stimulator used primarily for the treatment of pulmonary arterial hypertension and chronic thromboembolic pulmonary hypertension, as well as BAY60-2770, can rebuild LSEC fenestrae and reverse liver fibrosis by activating the downstream signaling pathway of NO [7,79].

### 5.3. Anti-Angiogenic Agents

As a critical pro-angiogenic factor, VEGF is of great significance in maintaining the phenotype of LSEC. However, overexpressed VEGF can lead to pathological angiogenesis and drive LSECs to undergo capillarization (Table 3).

Plumbagin is a naturally occurring naphthoquinone with significant potential in the anti-cancer, anti-inflammatory and anti-fibrotic fields. It can reverse LSEC capillarization by reducing VEGF expression [80]. However, its low solubility, lipophilicity, and short half-life characteristics limit its application [81]. To overcome its physicochemical limitations, plumbagin is often combined with nanocarriers, like liposomes and nanoparticles, enhancing its therapeutic efficacy and bioavailability [82].

Chito-oligosaccharides (COSs) are low-molecular-weight polymers derived from chitosan and are known for their diverse biological activities, including anti-fibrotic and anti-oxidative effects. They are recognized for their excellent solubility, biodegradability, and non-toxicity, which makes them superior to their parent compounds, chitin and chitosan [83,84]. However, COSs exhibit limited pharmacological efficacy owing to their poor oral bioavailability. Luckily, nano-encapsulation can markedly enhance the bioavailability of COSs. This approach suppresses angiogenesis through downregulation of VEGF-α and HIF-1α, reversing the capillarization of LSECs, and thereby demonstrating effective hepatoprotective and anti-fibrotic actions [85].

Carvedilol is a non-selective beta-blocker with additional alpha-1 adrenergic blocking properties, mainly used in cardiovascular disease. Carvedilol has been identified as a potential treatment for liver fibrosis by suppressing autophagy and promoting apoptosis in HSCs [86]. In addition, it can reduce the expression level of VEGF, effectively improve the structure of LSEC fenestrae, and mitigate liver fibrosis [87]. Alpha 1-adrenoceptor antagonist doxazosin has similar pharmacological effects and plays an important role in the treatment of liver fibrosis [88,89].

### 5.4. Actin-Modulating Agents

The activation of MK/Integrin α6/Src signaling pathway induces abnormal polymerization of actin, leading to alterations in the diameter and distribution of fenestrae during the process of LSEC defenestration. Addressing this pathological mechanism, studies have found that liquiritin, a flavonoid derived from licorice root with anti-inflammatory effects, exerts therapeutic effects on liver fibrosis. In detail, liquiritin inhibits the expression of MK and integrin α6, further reduces Src phosphorylation levels, and subsequently induces actin depolymerization, ultimately restoring fenestrae diameter and alleviating liver fibrosis [43,90]. Similarly, cytochalasin B also regulates the diameter and distribution of fenestrae by promoting the depolymerization of actin, reducing the formation of stress fibers and remodeling the cytoskeleton, therefore alleviating hepatic fibrosis [91] (Table 4).

### 5.5. Others

Notably, the targeted delivery efficiency of other anti-fibrotic drugs to the liver can also be effectively improved by restoring the LSEC fenestrae [92]. As a substrate of eNOS, L-arginine (L-Arg) has been shown to reconstruct LSEC fenestrae by promoting NO generation. Utilizing this mechanism, Li et al. successfully achieve the targeted delivery of all-trans retinoic acid to HSCs, overcoming the pathological barriers that hinder drug delivery in liver fibrosis treatment. Furthermore, the combination of riociguat and the small-molecule drug JQ-1 (a small-molecule inhibitor targeting the bromodomain and extra-terminal family of proteins involved in regulating gene expression), which is encapsulated in targeted nano-peptides, not only reduces drug toxicity but also markedly enhances the efficiency of targeted delivery to HSCs [79,93]. Moreover, atorvastatin encapsulated within neutrophil membrane liposomes can restore the fenestrated structure of LSECs in fibrotic liver areas. This is followed by the use of vitamin A as a functional molecule to bind with retinoid receptors, enabling the targeted delivery of albumin to activated HSCs for effective treatment of liver fibrosis [76]. These studies provide novel and promising strategies.

## 6. Conclusions and Outlook

The fenestrae of LSECs has an indispensable effect on substance exchange, immune regulation and hemodynamic regulation within the liver. The structural and functional integrity of these fenestrae is vital for supporting the normal physiological processes of the liver. However, the loss of fenestrae of LSECs represents a crucial pathological event in the onset and development of liver fibrosis. LSEC defenestration not only leads to disrupted substance exchange and hepatic microcirculation but also promotes the activation of HSCs, resulting in excessive ECM deposition through aberrant secretion and dysregulation of signaling pathways. These processes collectively drive the advancement of liver fibrosis.

In recent years, the significance of restoring the fenestrae structure of LSECs in the context of liver fibrosis treatment has garnered increasing attention. Restoration of fenestrae not only improves the liver microcirculation and inhibits the activation of HSCs but also addresses the issue of substance transport barriers in fibrosis treatment by enhancing drug delivery efficiency. Many drugs are showing promise as candidates for hepatic fibrosis treatment by regulating the fenestrae of LSECs. However, most of them exhibit poor solubility and low bioavailability, but this problem can be better solved through drug structural modification, changing crystal forms or pharmaceutical formulations. However, several limitations remain in current studies. Firstly, the molecular mechanisms underlying LSEC defenestration remain incompletely understood, especially in liver fibrosis caused by different etiologies, which requires further exploration. Secondly, existing potential treatment strategies are largely studied in animals, and their clinical translation remains challenging. Extensive clinical data are required to validate the safety and efficacy of these approaches. Additionally, many drugs possess multiple pharmacological effects, and improving their selectivity for liver fibrosis treatment remains a challenge.

## Figures and Tables

**Table 1:** Structures and mechanisms of drugs for treating LSEC defenestration and liver fibrosis through alleviating oxidative stress damage.

Drug	Skeletal Formula	Related Mechanism
Resveratrol	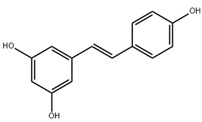	Inhibit NOX
N-acetylcysteine	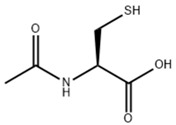	Supplement GSH
Curcumin	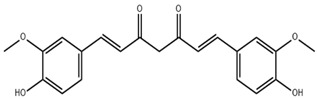	Inhibit NF-κB signaling pathway

**Table 2:** Structures and mechanisms of drugs for treating LSEC defenestration and liver fibrosis through activating eNOS/NO/sGC signaling pathway.

Drug	Skeletal Formula	Related Mechanism
Tofogliflozin	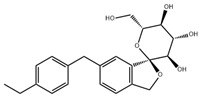	Upregulate eNOS expression and its phosphorylation levels
Sulodexide	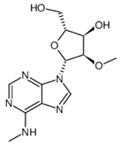	Upregulate eNOS expression
Atorvastatin	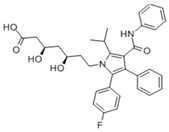	Increase the phosphorylation of eNOS
Metformin	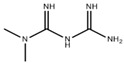	Upregulate eNOS expression
Riociguat	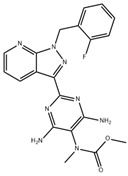	Active sGC receptor
BAY60-2770	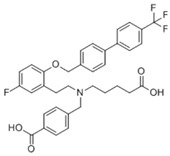	Active sGC receptor
L-arginine	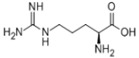	Promote NO generation, as a substrate of eNOS

**Table 3:** Structures and mechanisms of drugs for treating LSEC defenestration and liver fibrosis through inhibiting pathological angiogenesis.

Drug	Skeletal Formula	Related Mechanism
Plumbagin	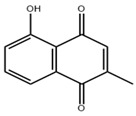	Reduce VEGF expression
Chitooligosaccharides	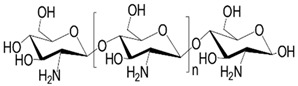	Reduce VEGF and HIF-1 expression
Carvedilol	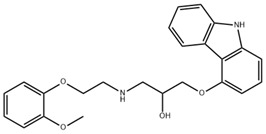	Reduce VEGF expression
Doxazosin	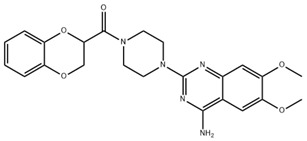	Reduce VEGF expression

**Table 4:** Structures and mechanisms of drugs for treating LSEC defenestration and liver fibrosis through modulating F-actin.

Drug	Skeletal Formula	Related Mechanism
Liquiritin	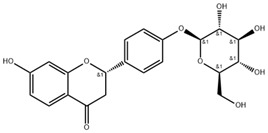	Inhibit the expression of MK and integrin α6, and reduce the Src phosphorylation levels
Cytochalasin B	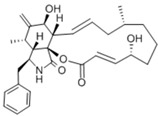	Depolymerize F-actin

## Data Availability

No new data were created or analyzed in this study. Data sharing is not applicable to this article.

## References

[1] Llovet J.M., Zucman-Rossi J., Pikarsky E., Sangro B., Schwartz M., Sherman M., Gores G. (2016). Hepatocellular carcinoma. Nat. Rev. Dis. Primers.

[2] Tao H., Liu Q., Zeng A., Song L. (2023). Unlocking the potential of Mesenchymal stem cells in liver Fibrosis: Insights into the impact of autophagy and aging. Int. Immunopharmacol..

[3] Roehlen N., Crouchet E., Baumert T.F. (2020). Liver Fibrosis: Mechanistic Concepts and Therapeutic Perspectives. Cells.

[4] Smedsrød B., Pertoft H., Gustafson S., Laurent T.C. (1990). Scavenger functions of the liver endothelial cell. Biochem. J..

[5] Cheng Q.N., Yang X., Wu J.F., Ai W.B., Ni Y.R. (2021). Interaction of non-parenchymal hepatocytes in the process of hepatic fibrosis (Review). Mol. Med. Rep..

[6] Bhandari S., Larsen A.K., McCourt P., Smedsrød B., Sørensen K.K. (2021). The Scavenger Function of Liver Sinusoidal Endothelial Cells in Health and Disease. Front. Physiol..

[7] Xie G., Wang X., Wang L., Wang L., Atkinson R.D., Kanel G.C., Gaarde W.A., Deleve L.D. (2012). Role of differentiation of liver sinusoidal endothelial cells in progression and regression of hepatic fibrosis in rats. Gastroenterology.

[8] Poisson J., Lemoinne S., Boulanger C., Durand F., Moreau R., Valla D., Rautou P.E. (2017). Liver sinusoidal endothelial cells: Physiology and role in liver diseases. J. Hepatol..

[9] Szafranska K., Kruse L.D., Holte C.F., McCourt P., Zapotoczny B. (2021). The wHole Story About Fenestrations in LSEC. Front. Physiol..

[10] Lafoz E., Ruart M., Anton A., Oncins A., Hernández-Gea V. (2020). The Endothelium as a Driver of Liver Fibrosis and Regeneration. Cells.

[11] Gracia-Sancho J., Caparrós E., Fernández-Iglesias A., Francés R. (2021). Role of liver sinusoidal endothelial cells in liver diseases. Nat. Rev. Gastroenterol. Hepatol..

[12] Sørensen K.K., Simon-Santamaria J., McCuskey R.S., Smedsrød B. (2015). Liver Sinusoidal Endothelial Cells. Compr. Physiol..

[13] He Q., He W., Dong H., Guo Y., Yuan G., Shi X., Wang D., Lu F. (2024). Role of liver sinusoidal endothelial cell in metabolic dysfunction-associated fatty liver disease. Cell Commun. Signal.

[14] Wang L., He L., Yi W., Wang M., Xu F., Liu H., Nie J., Pan Y.H., Dang S., Zhang W. (2024). ADAMTS18-fibronectin interaction regulates the morphology of liver sinusoidal endothelial cells. iScience.

[15] Furuta K., Tang X., Islam S., Tapia A., Chen Z.B., Ibrahim S.H. (2023). Endotheliopathy in the metabolic syndrome: Mechanisms and clinical implications. Pharmacol. Ther..

[16] Zhao S., Zhang L., Zhao J., Tasnim F., Yu H. (2024). Publication characteristics and visualized analysis of research about liver sinusoidal endothelial cells. iLIVER.

[17] Deleve L.D., Wang X., Guo Y. (2008). Sinusoidal endothelial cells prevent rat stellate cell activation and promote reversion to quiescence. Hepatology.

[18] Fraser R., Dobbs B.R., Rogers G.W. (1995). Lipoproteins and the liver sieve: The role of the fenestrated sinusoidal endothelium in lipoprotein metabolism, atherosclerosis, and cirrhosis. Hepatology.

[19] Parmar K.M., Larman H.B., Dai G., Zhang Y., Wang E.T., Moorthy S.N., Kratz J.R., Lin Z., Jain M.K., Gimbrone M.A. (2006). Integration of flow-dependent endothelial phenotypes by Kruppel-like factor 2. J. Clin. Investig..

[20] Zhang Q., Liu J., Liu J., Huang W., Tian L., Quan J., Wang Y., Niu R. (2014). oxLDL induces injury and defenestration of human liver sinusoidal endothelial cells via LOX1. J. Mol. Endocrinol..

[21] Cogger V.C., Mohamad M., Solon-Biet S.M., Senior A.M., Warren A., O’Reilly J.N., Tung B.T., Svistounov D., McMahon A.C., Fraser R. (2016). Dietary macronutrients and the aging liver sinusoidal endothelial cell. Am. J. Physiol. Heart Circ. Physiol..

[22] Deaciuc I.V., D’Souza N.B., Sarphie T.G., Schmidt J., Hill D.B., McClain C.J. (1999). Effects of exogenous superoxide anion and nitric oxide on the scavenging function and electron microscopic appearance of the sinusoidal endothelium in the isolated, perfused rat liver. J. Hepatol..

[23] Straub A.C., Clark K.A., Ross M.A., Chandra A.G., Li S., Gao X., Pagano P.J., Stolz D.B., Barchowsky A. (2008). Arsenic-stimulated liver sinusoidal capillarization in mice requires NADPH oxidase-generated superoxide. J. Clin. Investig..

[24] Dobbs B.R., Rogers G.W., Xing H.Y., Fraser R. (1994). Endotoxin-induced defenestration of the hepatic sinusoidal endothelium: A factor in the pathogenesis of cirrhosis?. Liver.

[25] Braet F., Wisse E. (2002). Structural and functional aspects of liver sinusoidal endothelial cell fenestrae: A review. Comp. Hepatol..

[26] Hilmer S.N., Cogger V.C., Fraser R., McLean A.J., Sullivan D., Le Couteur D.G. (2005). Age-related changes in the hepatic sinusoidal endothelium impede lipoprotein transfer in the rat. Hepatology.

[27] Mohamad M., Mitchell S.J., Wu L.E., White M.Y., Cordwell S.J., Mach J., Solon-Biet S.M., Boyer D., Nines D., Das A. (2016). Ultrastructure of the liver microcirculation influences hepatic and systemic insulin activity and provides a mechanism for age-related insulin resistance. Aging Cell.

[28] Zhang X., Li P., Zhou J., Zhang Z., Wu H., Shu X., Li W., Wu Y., Du Y., Lü D. (2024). FAK-p38 signaling serves as a potential target for reverting matrix stiffness-modulated liver sinusoidal endothelial cell defenestration. Biomaterials.

[29] Mitchell S.J., Huizer-Pajkos A., Cogger V.C., McLachlan A.J., Le Couteur D.G., Jones B., de Cabo R., Hilmer S.N. (2011). Age-related pseudocapillarization of the liver sinusoidal endothelium impairs the hepatic clearance of acetaminophen in rats. J. Gerontol. A Biol. Sci. Med. Sci..

[30] Kaur S., Anita K. (2013). Angiogenesis in liver regeneration and fibrosis: “A double-edged sword”. Hepatol. Int..

[31] Duan J.L., Ruan B., Yan X.C., Liang L., Song P., Yang Z.Y., Liu Y., Dou K.F., Han H., Wang L. (2018). Endothelial Notch activation reshapes the angiocrine of sinusoidal endothelia to aggravate liver fibrosis and blunt regeneration in mice. Hepatology.

[32] Kruse L.D., Holte C., Zapotoczny B., Struck E.C., Schürstedt J., Hübner W., Huser T., Szafranska K. (2025). Hydrogen peroxide damage to rat liver sinusoidal endothelial cells is prevented by n-acetyl-cysteine but not GSH. Hepatol. Commun..

[33] Cogger V.C., Muller M., Fraser R., McLean A.J., Khan J., Le Couteur D.G. (2004). The effects of oxidative stress on the liver sieve. J. Hepatol..

[34] Francque S., Laleman W., Verbeke L., Van Steenkiste C., Casteleyn C., Kwanten W., Van Dyck C., D’Hondt M., Ramon A., Vermeulen W. (2012). Increased intrahepatic resistance in severe steatosis: Endothelial dysfunction, vasoconstrictor overproduction and altered microvascular architecture. Lab. Investig..

[35] Luo X., Dan W., Luo X., Zhu X., Wang G., Ning Z., Li Y., Ma X., Yang R., Jin S. (2017). Caveolin 1-related autophagy initiated by aldosterone-induced oxidation promotes liver sinusoidal endothelial cells defenestration. Redox Biol..

[36] Maslak E., Gregorius A., Chlopicki S. (2015). Liver sinusoidal endothelial cells (LSECs) function and NAFLD; NO-based therapy targeted to the liver. Pharmacol. Rep..

[37] Mittal M., Siddiqui M.R., Tran K., Reddy S.P., Malik A.B. (2014). Reactive oxygen species in inflammation and tissue injury. Antioxid. Redox Signal.

[38] Desroches-Castan A., Tillet E., Ricard N., Ouarné M., Mallet C., Feige J.J., Bailly S. (2019). Differential Consequences of *Bmp9* Deletion on Sinusoidal Endothelial Cell Differentiation and Liver Fibrosis in 129/Ola and C57BL/6 Mice. Cells.

[39] He S., Luo Y., Ma W., Wang X., Yan C., Hao W., Fang Y., Su H., Lai B., Liu J. (2024). Endothelial POFUT1 controls injury-induced liver fibrosis by repressing fibrinogen synthesis. J. Hepatol..

[40] Venkatraman L., Tucker-Kellogg L. (2013). The CD47-binding peptide of thrombospondin-1 induces defenestration of liver sinusoidal endothelial cells. Liver Int..

[41] Zhong Y., Xu M., Hu J., Huang X., Lin N., Deng M. (2021). Inhibiting Th1/2 cells influences hepatic capillarization by adjusting sinusoidal endothelial fenestrae through Rho-ROCK-myosin pathway. Aging.

[42] Czyzynska-Cichon I., Kotlinowski J., Blacharczyk O., Giergiel M., Szymanowski K., Metwally S., Wojnar-Lason K., Dobosz E., Koziel J., Lekka M. (2024). Early and late phases of liver sinusoidal endothelial cell (LSEC) defenestration in mouse model of systemic inflammation. Cell Mol. Biol. Lett..

[43] Wu L., Chen H., Fu C., Xing M., Fang H., Yang F., Yang Q., Zhang Y., Li W., Chen Z. (2022). Midkine mediates dysfunction of liver sinusoidal endothelial cells through integrin α4 and α6. Vasc. Pharmacol..

[44] Urschel K., Garlichs C.D., Daniel W.G., Cicha I. (2011). VEGFR2 signalling contributes to increased endothelial susceptibility to TNF-α under chronic non-uniform shear stress. Atherosclerosis.

[45] Xue X., Zhao X., Wang J., Wang C., Ma C., Zhang Y., Li Y., Peng C. (2023). Carthami flos extract against carbon tetrachloride-induced liver fibrosis via alleviating angiogenesis in mice. Phytomedicine.

[46] Rosmorduc O., Housset C. (2010). Hypoxia: A link between fibrogenesis, angiogenesis, and carcinogenesis in liver disease. Semin. Liver Dis..

[47] Bocca C., Novo E., Miglietta A., Parola M. (2015). Angiogenesis and Fibrogenesis in Chronic Liver Diseases. Cell Mol. Gastroenterol. Hepatol..

[48] Wang Y., Wang C., Yang F., Chen Y., Shi Y., Xu R., Zhang Z., Yan Y. (2024). USP9X-enriched MSC-sEV inhibits LSEC angiogenesis in MASH mice by downregulating the IκBα/NF-κB/Ang-2 pathway. Pharmacol. Res..

[49] Mori T., Okanoue T., Sawa Y., Hori N., Ohta M., Kagawa K. (1993). Defenestration of the sinusoidal endothelial cell in a rat model of cirrhosis. Hepatology.

[50] Rogers G.W.T., Dobbs B.R., Fraser R. (1993). Defenestration of the Hepatic Sinusoidal Endothelium in the Dimethylnitrosamine fed Rat: Is this process Reversible?. Cells Hepatic Sinusoid.

[51] Winkler M., Staniczek T., Kürschner S.W., Schmid C.D., Schönhaber H., Cordero J., Kessler L., Mathes A., Sticht C., Neßling M. (2021). Endothelial GATA4 controls liver fibrosis and regeneration by preventing a pathogenic switch in angiocrine signaling. J. Hepatol..

[52] Babbs C., Haboubi N.Y., Mellor J.M., Smith A., Rowan B.P., Warnes T.W. (1990). Endothelial cell transformation in primary biliary cirrhosis: A morphological and biochemical study. Hepatology.

[53] Wisse E., De Zanger R.B., Charels K., Van Der Smissen P., McCuskey R.S. (1985). The liver sieve: Considerations concerning the structure and function of endothelial fenestrae, the sinusoidal wall and the space of Disse. Hepatology.

[54] Tanoi T., Tamura T., Sano N., Nakayama K., Fukunaga K., Zheng Y.W., Akhter A., Sakurai Y., Hayashi Y., Harashima H. (2016). Protecting liver sinusoidal endothelial cells suppresses apoptosis in acute liver damage. Hepatol. Res..

[55] Golse N., Bucur P.O., Adam R., Castaing D., Sa Cunha A., Vibert E. (2013). New paradigms in post-hepatectomy liver failure. J. Gastrointest. Surg..

[56] DeLeve L.D. (2015). Liver sinusoidal endothelial cells in hepatic fibrosis. Hepatology.

[57] Feder L.S., Todaro J.A., Laskin D.L. (1993). Characterization of interleukin-1 and interleukin-6 production by hepatic endothelial cells and macrophages. J. Leukoc. Biol..

[58] Connolly M.K., Bedrosian A.S., Malhotra A., Henning J.R., Ibrahim J., Vera V., Cieza-Rubio N.E., Hassan B.U., Pachter H.L., Cohen S. (2010). In hepatic fibrosis, liver sinusoidal endothelial cells acquire enhanced immunogenicity. J. Immunol..

[59] Neubauer K., Krüger M., Quondamatteo F., Knittel T., Saile B., Ramadori G. (1999). Transforming growth factor-β1 stimulates the synthesis of basement membrane proteins laminin, collagen type IV and entactin in rat liver sinusoidal endothelial cells. J. Hepatol..

[60] de la Lastra C.A., Villegas I. (2007). Resveratrol as an antioxidant and pro-oxidant agent: Mechanisms and clinical implications. Biochem. Soc. Trans..

[61] Luo X., Bai Y., He S., Sun S., Jiang X., Yang Z., Lu D., Wei P., Liang Y., Peng C. (2021). Sirtuin 1 ameliorates defenestration in hepatic sinusoidal endothelial cells during liver fibrosis via inhibiting stress-induced premature senescence. Cell Prolif..

[62] Pannu N., Bhatnagar A. (2019). Resveratrol: From enhanced biosynthesis and bioavailability to multitargeting chronic diseases. Biomed. Pharmacother..

[63] Zhitkovich A. (2019). N-Acetylcysteine: Antioxidant, Aldehyde Scavenger, and More. Chem. Res. Toxicol..

[64] Pereira-Filho G., Ferreira C., Schwengber A., Marroni C., Zettler C., Marroni N. (2008). Role of N-acetylcysteine on fibrosis and oxidative stress in cirrhotic rats. Arq. Gastroenterol..

[65] Sun Y., Pu L.Y., Lu L., Wang X.H., Zhang F., Rao J.H. (2014). N-acetylcysteine attenuates reactive-oxygen-species-mediated endoplasmic reticulum stress during liver ischemia-reperfusion injury. World J. Gastroenterol..

[66] Lee P.C., Yang Y.Y., Huang C.S., Hsieh S.L., Lee K.C., Hsieh Y.C., Lee T.Y., Lin H.C. (2015). Concomitant inhibition of oxidative stress and angiogenesis by chronic hydrogen-rich saline and N-acetylcysteine treatments improves systemic, splanchnic and hepatic hemodynamics of cirrhotic rats. Hepatol. Res..

[67] Zheng Y., Wang J., Wang J., Xie H., Zhao T. (2020). Effect of Curcumol on the Fenestrae of Liver Sinusoidal Endothelial Cells Based on NF-κB Signaling Pathway. Evid. Based Complement. Altern. Med..

[68] Yen F.L., Wu T.H., Tzeng C.W., Lin L.T., Lin C.C. (2010). Curcumin nanoparticles improve the physicochemical properties of curcumin and effectively enhance its antioxidant and antihepatoma activities. J. Agric. Food Chem..

[69] Rodrigues F.C., Kumar N.A., Thakur G. (2021). The potency of heterocyclic curcumin analogues: An evidence-based review. Pharmacol. Res..

[70] Asada S., Kaji K., Nishimura N., Koizumi A., Matsuda T., Tanaka M., Yorioka N., Sato S., Kitagawa K., Namisaki T. (2024). Tofogliflozin Delays Portal Hypertension and Hepatic Fibrosis by Inhibiting Sinusoidal Capillarization in Cirrhotic Rats. Cells.

[71] Veraldi N., Guerrini M., Urso E., Risi G., Bertini S., Bensi D., Bisio A. (2018). Fine structural characterization of sulodexide. J. Pharm. Biomed. Anal..

[72] Dauth A., Bręborowicz A., Ruan Y., Tang Q., Zadeh J.K., Böhm E.W., Pfeiffer N., Khedkar P.H., Patzak A., Vujacic-Mirski K. (2023). Sulodexide Prevents Hyperglycemia-Induced Endothelial Dysfunction and Oxidative Stress in Porcine Retinal Arterioles. Antioxidants.

[73] Huang R., Deng J., Zhu C.P., Liu S.Q., Cui Y.L., Chen F., Zhang X., Tao X., Xie W.F. (2023). Sulodexide attenuates liver fibrosis in mice by restoration of differentiated liver sinusoidal endothelial cell. Biomed. Pharmacother..

[74] Ghoreshi Z.A., Kabirifar R., Khodarahmi A., Karimollah A., Moradi A. (2020). The preventive effect of atorvastatin on liver fibrosis in the bile duct ligation rats via antioxidant activity and down-regulation of Rac1 and NOX1. Iran. J. Basic Med. Sci..

[75] Alnajjar R., Mohamed N., Kawafi N. (2021). Bicyclo[1.1.1]Pentane as Phenyl Substituent in Atorvastatin Drug to improve Physicochemical Properties: Drug-likeness, DFT, Pharmacokinetics, Docking, and Molecular Dynamic Simulation. J. Mol. Struct..

[76] Sun D., Du X., Cao X., Wu B., Li S., Zhao Y., Liu T., Xu L., Huang H. (2024). Neutrophil-Based Bionic Delivery System Breaks Through the Capillary Barrier of Liver Sinusoidal Endothelial Cells and Inhibits the Activation of Hepatic Stellate Cells. Mol. Pharm..

[77] Su Y., Lu S., Hou C., Ren K., Wang M., Liu X., Zhao S., Liu X. (2022). Mitigation of liver fibrosis via hepatic stellate cells mitochondrial apoptosis induced by metformin. Int. Immunopharmacol..

[78] Hunt N.J., Lockwood G.P., Kang S.W.S., Pulpitel T., Clark X., Mao H., McCourt P.A.G., Cooney G.J., Wali J.A., Le Couteur F.H. (2020). The Effects of Metformin on Age-Related Changes in the Liver Sinusoidal Endothelial Cell. J. Gerontol. A Biol. Sci. Med. Sci..

[79] Li F., Zhao Y., Cheng Z., Wang Y., Yue Y., Cheng X., Sun J., Atabakhshi-Kashi M., Yao J., Dou J. (2023). Restoration of Sinusoid Fenestrae Followed by Targeted Nanoassembly Delivery of an Anti-Fibrotic Agent Improves Treatment Efficacy in Liver Fibrosis. Adv. Mater..

[80] Li G., Peng Y., Zhao T., Lin J., Duan X., Wei Y., Ma J. (2017). Plumbagin Alleviates Capillarization of Hepatic Sinusoids In Vitro by Downregulating ET-1, VEGF, LN, and Type IV Collagen. Biomed. Res. Int..

[81] Ahmad I., Tabrez S. (2024). Exploring natural resources: Plumbagin as a potent anticancer agent. S. Afr. J. Bot..

[82] Tripathi S.K., Panda M., Biswal B.K. (2019). Emerging role of plumbagin: Cytotoxic potential and pharmaceutical relevance towards cancer therapy. Food Chem. Toxicol..

[83] Phil L., Naveed M., Mohammad I.S., Bo L., Bin D. (2018). Chitooligosaccharide: An evaluation of physicochemical and biological properties with the proposition for determination of thermal degradation products. Biomed. Pharmacother..

[84] Liaqat F., Eltem R. (2018). Chitooligosaccharides and their biological activities: A comprehensive review. Carbohydr. Polym..

[85] Liu P., Li H., Li R., Geng Y., Gong J., Xu H., Xu Z., Shi J. (2022). Nanoencapsulation of chitooligosaccharides enhances its oral bioavailability and anti-liver fibrotic effects. Food Res. Int..

[86] Meng D., Li Z., Wang G., Ling L., Wu Y., Zhang C. (2018). Carvedilol attenuates liver fibrosis by suppressing autophagy and promoting apoptosis in hepatic stellate cells. Biomed. Pharmacother..

[87] Wu Y., Li Z., Xiu A.Y., Meng D.X., Wang S.N., Zhang C.Q. (2019). Carvedilol attenuates carbon tetrachloride-induced liver fibrosis and hepatic sinusoidal capillarization in mice. Drug Des. Dev. Ther..

[88] Xiu A.Y., Ding Q., Zhu C.P., Zhang C.Q. (2024). The α-1 Adrenergic Receptor Antagonist Doxazosin Attenuates Liver Fibrosis by Alleviating Sinusoidal Capillarization and Liver Angiogenesis. Adv. Biol..

[89] Xiu A.Y., Ding Q., Li Z., Zhang C.Q. (2021). Doxazosin Attenuates Liver Fibrosis by Inhibiting Autophagy in Hepatic Stellate Cells via Activation of the PI3K/Akt/mTOR Signaling Pathway. Drug Des. Devel Ther..

[90] Fu C., Zhang Y., Xi W.J., Xu K., Meng F., Ma T., Li W., Wu L., Chen Z. (2023). Dahuang Zhechong pill attenuates hepatic sinusoidal capillarization in liver cirrhosis and hepatocellular carcinoma rat model via the MK/integrin signaling pathway. J. Ethnopharmacol..

[91] Di Martino J., Mascalchi P., Legros P., Lacomme S., Gontier E., Bioulac-Sage P., Balabaud C., Moreau V., Saltel F. (2019). Actin Depolymerization in Dedifferentiated Liver Sinusoidal Endothelial Cells Promotes Fenestrae Re-Formation. Hepatol. Commun..

[92] Meng X., Zhu G., Yang Y.G., Sun T. (2024). Targeted delivery strategies: The interactions and applications of nanoparticles in liver diseases. Biomed. Pharmacother..

[93] Wang K., Chen H., Zheng J., Chen J., Chen Y., Yuan Y. (2024). Engineered liposomes targeting hepatic stellate cells overcome pathological barriers and reverse liver fibrosis. J. Control Release.

